# Clofarabine monotherapy in two patients with refractory Langerhans cell histiocytosis

**DOI:** 10.1002/cnr2.1579

**Published:** 2021-11-01

**Authors:** Masahiro Irie, Tomohiro Nakano, Saori Katayama, Tasuku Suzuki, Kunihiko Moriya, Yuko Watanabe, Nobu Suzuki, Yuka Saitoh‐Nanjyo, Masaei Onuma, Takeshi Rikiishi, Hidetaka Niizuma, Yoji Sasahara, Shigeo Kure

**Affiliations:** ^1^ Department of Pediatrics Tohoku University Graduate School of Medicine Sendai Japan; ^2^ Department of Pediatric Oncology National Cancer Center Hospital Chuo‐ku Japan; ^3^ Department of Hematology and Oncology Miyagi Children's Hospital Sendai Japan

**Keywords:** allogeneic hematopoietic stem cell transplantation, cladribine, clofarabine, high dose‐cytarabine, refractory Langerhans cell histiocytosis

## Abstract

**Background:**

Better therapeutic options other than conventional chemotherapy for pediatric patients with refractory Langerhans cell histiocytosis (LCH) remain undetermined.

**Case:**

We successfully treated two patients with refractory and risk organ negative LCH with clofarabine (CLO) monotherapy after recurrence. We administered total 23 courses of CLO monotherapy in patient 1 and 4 courses in patient 2. Both patients had distinct clinical manifestations but achieved a durable complete response with acceptable adverse effects of transient myelosuppression. CLO monotherapy was still effective when he had the second recurrent lesion after first completion of CLO in patient 1. We could discontinue prednisolone to control his refractory inflammation of LCH after completing CLO chemotherapy in patient 2.

**Conclusion:**

Although large‐scale studies are warranted, CLO monotherapy could be a therapeutic option for high efficacy and feasibility besides other intensive combination chemotherapies or allogeneic hematopoietic stem cell transplantation for refractory LCH without risk organ involvement in children.

## 1 INTRODUCTION

Langerhans cell histiocytosis (LCH) is a rare and inflammatory neoplastic disease of bone marrow‐derived dendritic cells, which express CD1a and CD207 (langerin) along with mixed inflammatory cell infiltration.^1^ In general, patients with LCH have a good prognosis, and the 5‐year overall survival (OS) is >85%. However, patients with risk organs (liver, spleen, lung, and bone marrow) have worse prognoses.^2^, ^3^ Treatment for refractory multisystem LCH (LCH‐MS) is challenging. Some patients were treated with cladribine (2‐chlorodeoxyadenosine, 2‐CdA) and/or cytarabine (Ara‐C), but durable responses were inconsistent.^4^, ^5^, ^6^ Furthermore, intensive therapeutic regimens, such as combined 2‐CdA and high dose (HD)‐Ara‐C, are associated with significant acute toxicity and delayed immune reconstitution.^6^, ^7^ Some reports showed that clofarabine (CLO) monotherapy is effective for refractory LCH.^8^, ^9^

Herein, we report about two patients with refractory and risk organ negative LCH treated with repeated CLO monotherapy and achieved durable complete response with no severe adverse effects except for myelosuppression after recurrence.

## 2 CASES

### 2.1 Patient 1

**FIGURE 1:**
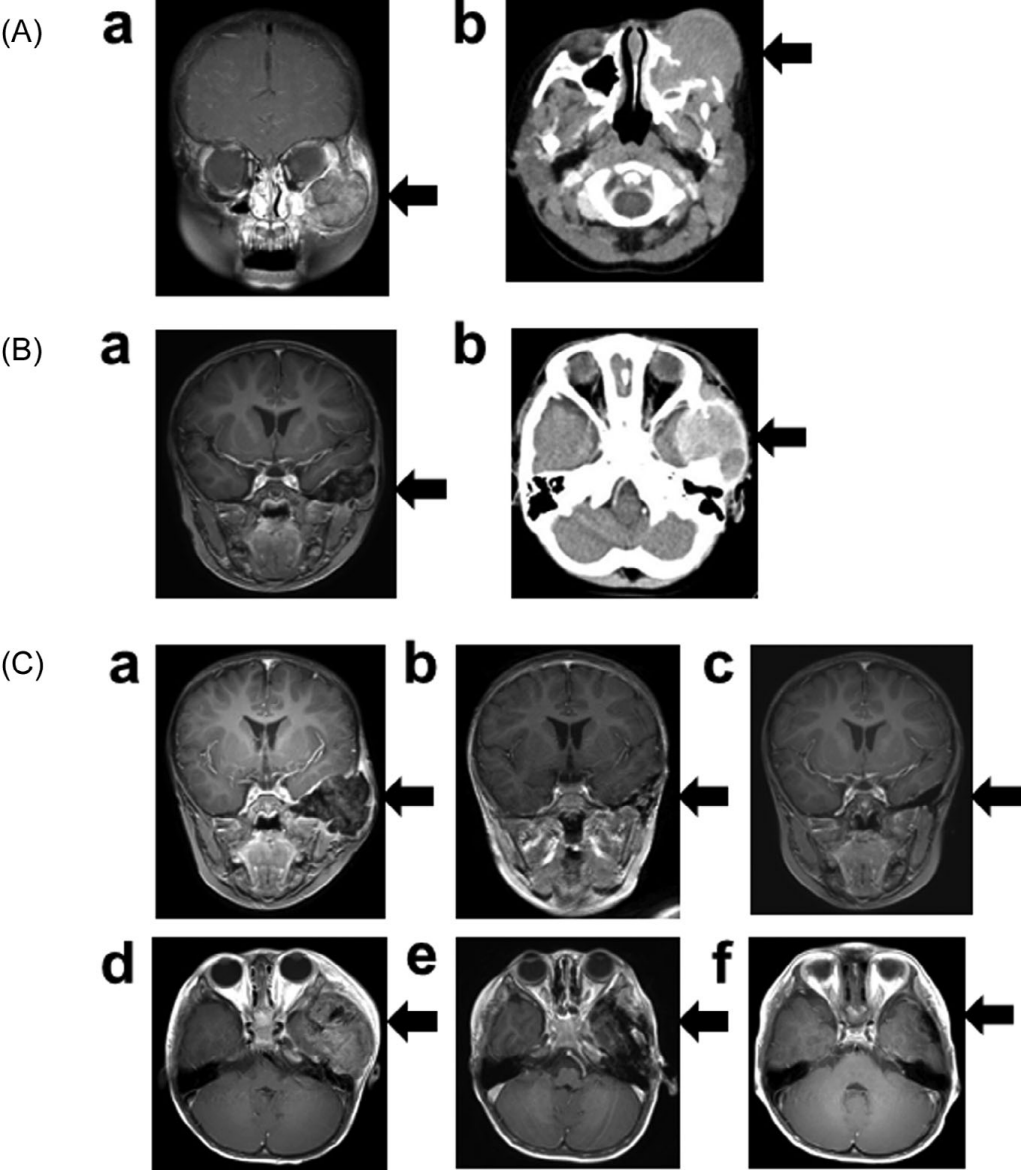
Clinical presentation of patient 1 showing primary and recurrent sites of LCH that were resolved by first CLO monotherapy. (A) The primarily involved sites of the left lower orbital wall and the maxillary sinus are shown by (a) gadolinium (Gd)‐enhanced magnetic resonance imaging (MRI) and (b) enhanced computed tomography (CT) axial images. (B) The recurrent lesion of the left sphenoidal bone is shown by (a) Gd‐enhanced MRI and (b) enhanced CT images. (C) Clinical response to CLO monotherapy is shown by Gd‐enhanced MRI (a) and (d) before treatment, (b) and (e) after two cycles of CLO, and (c) and (f) after the completion of 12 cycles of CLO. Arrows indicate each site of involved lesions

**FIGURE 2:**
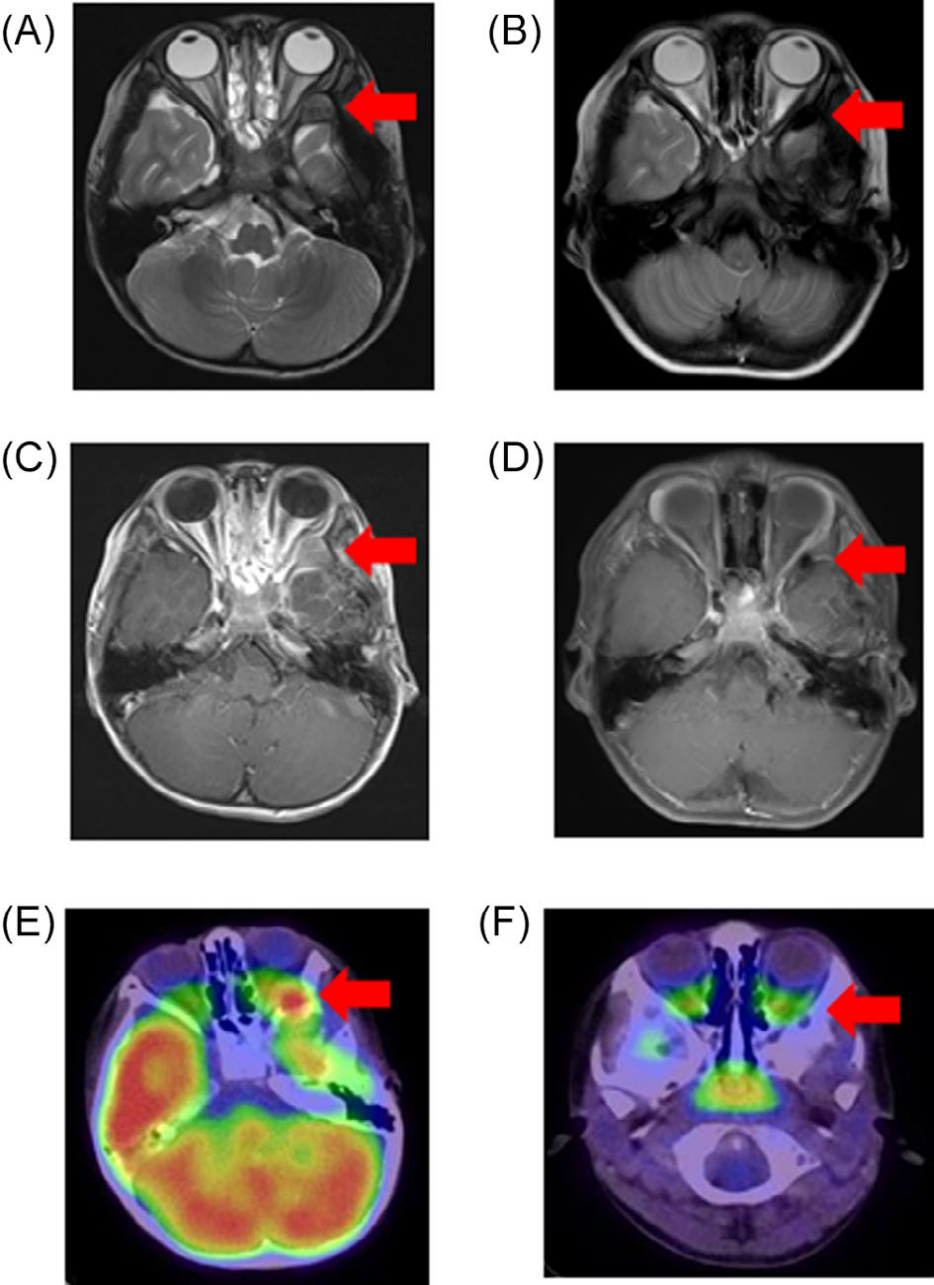
Clinical response to the second administration of CLO at post‐CLO recurrent site in patient 1. Second recurrent site after first completion of CLO is shown by (a) T2‐weighted MRI, (c) T1‐weighted MRI, (e) FDG‐PET before treatment and (b) T2‐weighted MRI, (d) T1‐weighted MRI, (f) FDG‐PET after second administration of six cycles of CLO. Red arrows indicate each site of involved lesions

A 3‐year‐old boy was diagnosed with LCH‐MS with periorbital bone and multiple skin lesions at 2 years of age (Figure 1A). He was initially treated with multidrug chemotherapy according to the Japanese LCH study group (JLSG)‐02 protocol.^10^ He achieved partial response, with a >50% decreased tumor volume after 6‐week induction chemotherapy consisting of prednisolone (PSL), vincristine (VCR), and Ara‐C. During subsequent maintenance therapy, he had a recurrence with a new skull lesion near the primary site 1 year after diagnosis (Figure 1B). A combination of PSL, cyclophosphamide (CPM), VCR, and doxorubicin (DOX) was administered as reinduction chemotherapy according to the JLSG‐02 protocol, which failed to reduce the tumor volume. Subsequent 2‐CdA monotherapy, administered as the third‐line chemotherapy, also failed to induce tumor regression. His skull lesion showed increasing growth, forming a large mass with osteolysis. It infiltrated into the epidural region, compressing his left temporal lobe of the brain during salvage therapies (Figure 1C, (a) and (d)). Consequently, he received CLO at a dose of 25 mg/m^2^/day for 5 days as the fourth‐line chemotherapy. The tumor size decreased after two cycles of CLO monotherapy (Figure 1C, (b) and (e)). The patient received 12 cycles of CLO and finally achieved a complete response (Figure 1C, (c) and (f)). During the follow‐up period, the LCH relapsed twice with intervals of 10 and 20 months; however, his new lesions also completely responded to a total of six and five courses of CLO monotherapy, respectively (Figure 2). He has been disease‐free for 2 years. During 23 cycles of CLO monotherapy, he experienced drug fever and capillary leak syndrome in the first cycle, which were later well controlled by PSL at a dose of 1 mg/kg/day administered for a week. The patient had myelosuppression with severe neutropenia (neutrophil counts <100/mm^3^), anemia, and thrombocytopenia, which resolved with granulocyte colony‐stimulating factor (G‐CSF), red blood cell concentrate (RBC) transfusion, and platelet concentrate (PC) transfusion. There were no life‐threatening infections such as sepsis and fungal pneumonia during any cycles of CLO monotherapy.

### 2.2 Patient 2

**FIGURE 3:**
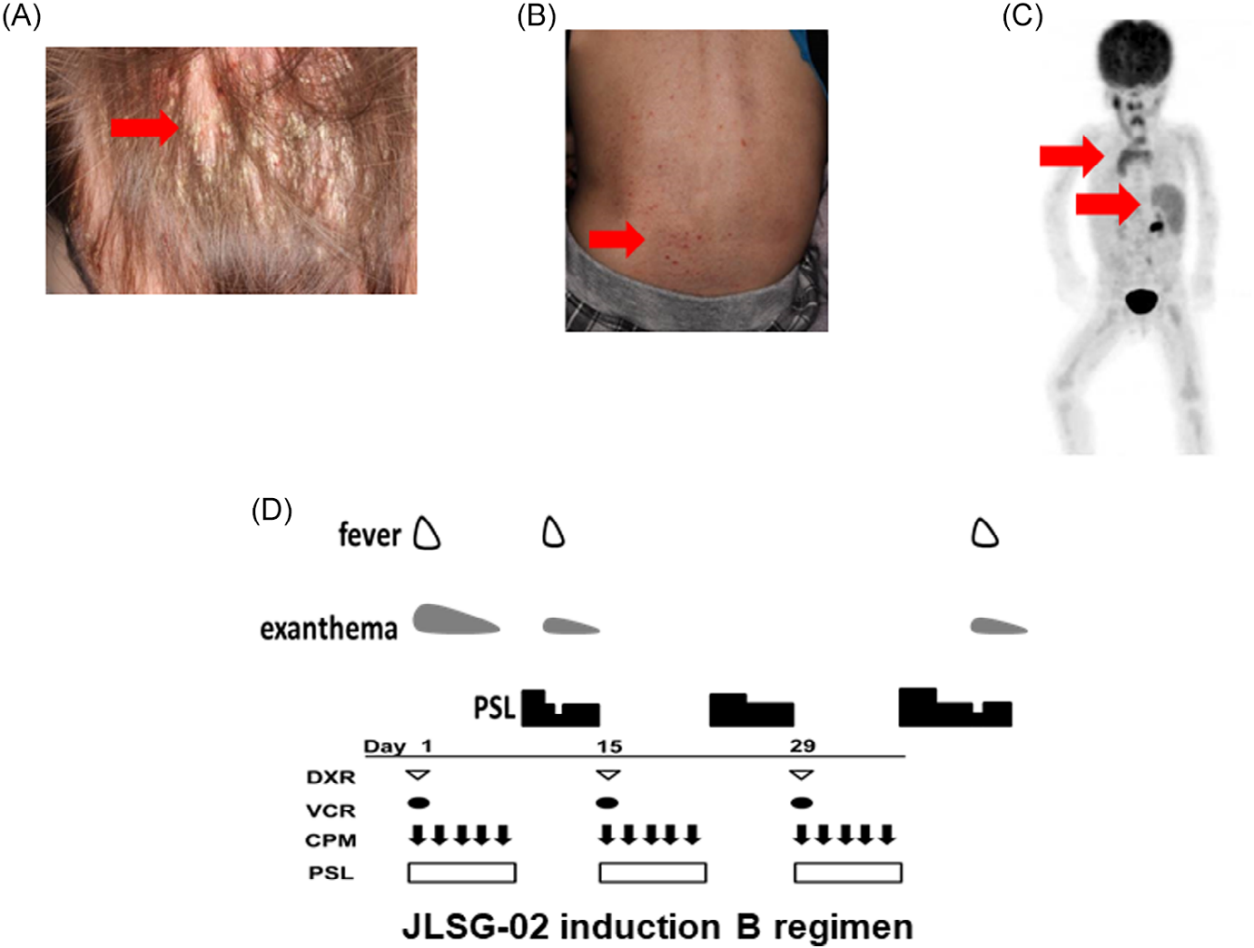
Clinical presentation of patient 2 showing recurrent sites of LCH. (A) The skin lesion resembling seborrheic dermatitis on his head, (B) The skin lesion resembled petechiae on his trunk, which revealed LCH reactivation by skin biopsy on recurrence. (C) Thymic and splenic inflammatory cell activation was revealed by FDG‐PET. (D) LCH reactivation symptoms, including high fever and skin lesions, were not controlled by JLSG‐02 induction B regimen. Red arrows indicate each site of involved lesions

**FIGURE 4:**
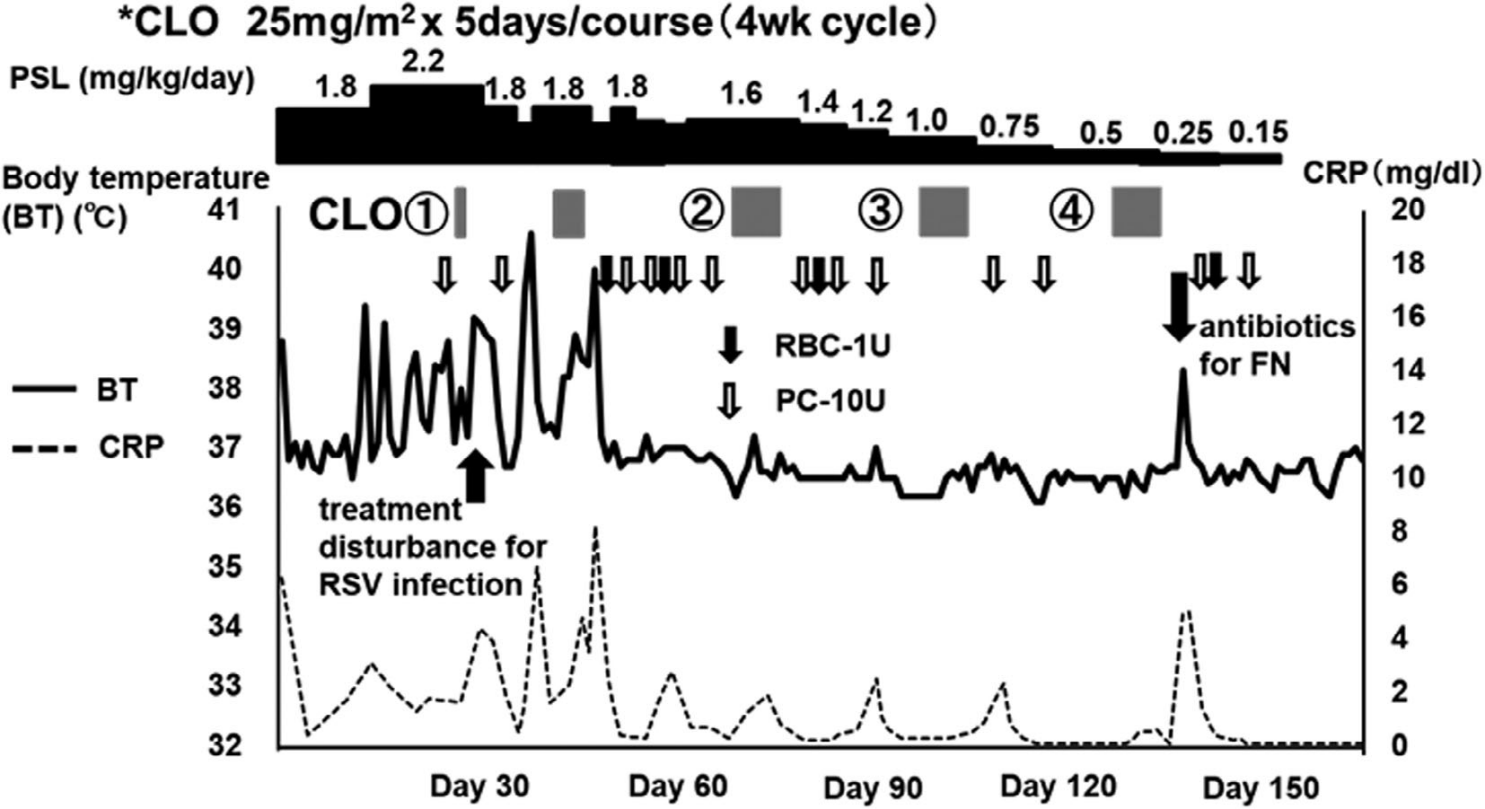
Clinical symptoms of patient 2 were resolved by CLO monotherapy. The recurrent high fever and PSL dependency regressed gradually during four courses of CLO monotherapy

A 2‐year‐old boy was diagnosed with single‐system multisite LCH (LCH‐SM, skin lesions alone) at 6 months of age, which developed to LCH‐MS at 1 year of age. He received 6‐week induction chemotherapy of PSL, VCR, and Ara‐C according to the JLSG‐02 protocol and achieved a partial response. During the maintenance therapy, he developed recurrent fever and abnormal skin lesions (Figure 3A,B). A skin biopsy revealed active LCH disease. Fluorodeoxyglucose‐positron emission tomography (FDG‐PET) revealed positivity in the thymus and spleen by activated inflammatory cells (Figure 3C). The reinduction chemotherapy (Induction B), consisting of PSL, VCR, CPM, and DOX according to the JLSG‐02 protocol,^10^ did not resolve his inflammatory symptoms. He required treatment with PSL continuously at a dose of >2 mg/kg/day (Figure 3D). Accordingly, patient 2 received the same dose of CLO used for patient 1 as third‐line chemotherapy. PSL at a dose of 0.5–1.8 mg/kg/day was administered in the same manner for the prophylaxis of drug fever and capillary leak syndrome. His symptoms resolved during the first cycle, and we reduced the PSL dose to 1 mg/kg/day after two cycles of CLO. We discontinued PSL after completing four CLO cycles, and he has been disease‐free for more than 3 years (Figure 4). He did not develop any severe adverse effects other than myelosuppression.

## 3 DISCUSSION

Patients with LCH generally have good prognoses, and the 5‐year OS is >85% even in patients with LCH‐MS and risk organ involvement.^8^ However, there are significant therapeutic problems in controlling disease activity without causing any life‐threatening adverse effects and long‐term sequelae in refractory and/or recurrent LCH‐MS. Herein, we described two patients with refractory LCH‐MS without risk organ involvement, both of whom were successfully treated with CLO monotherapy and achieved durable complete remission. Although patient 1 had recurrence twice after CLO treatment, he was also treated with CLO monotherapy and again achieved complete response. Although the clinical manifestations were distinct, CLO monotherapy was effective in both patients.

CLO is a second‐generation purine analog resembling cladribine and fludarabine in structure, except for its biochemical modifications that enhance its resistance to deamination and degradation.^8^ CLO is converted into the active form CLO 5′‐triphosphate in vivo and inhibits DNA polymerase activity and ribonucleotide reductase. The reported adverse effects of CLO include myelosuppression, capillary leak syndrome, headache, and peripheral neuropathy. In our two patients, myelosuppression, including grade 4 neutropenia, was frequently observed after CLO monotherapy, but it was well‐managed using G‐CSF, RBC, and PC transfusion and prophylactic antibiotics and antifungal agents. Capillary leak syndrome with fever, facial edema, and rash occurred during the first cycle of CLO monotherapy in patient 1, which were resolved by intravenous PSL administration. No other severe adverse events occurred after a total of 27 cycles of CLO monotherapy.

Simko et al. reported that among 11 patients with refractory or recurrent LCH, 10 responded to CLO monotherapy. The estimated progression‐free survival and OS at 1 year were 76% and 91%, respectively. There were no life‐threatening adverse effects during therapy in their study.^8^ The remaining issue is higher cost relative to other established chemotherapy options such as VCR/Ara‐C or 2‐CdA monotherapy. In a multicenter phase 2 study, Donadieu et al. reported that the combination of 2‐CdA and high‐dose Ara‐C effectively treated refractory LCH with risk organ involvement, with the 5‐year OS being 85% in 27 enrolled patients. However, six patients developed severe infections in their study, and two patients died due to treatment‐related toxicity. Therefore, substantially increased risk of mortality should be considered in patients with risk organ involvement and increased risk of toxicity from 2‐CdA and high‐dose Ara‐C treatment is warranted in this group.^11^ Moreover, in a retrospective analysis of 87 patients with refractory LCH treated with allogeneic hematopoietic stem cell transplantation (HSCT), Veys et al. reported that three out of four patients achieved long‐term event‐free survival. However, the transplant‐related mortality after HSCT was as high as 20%–30%.^12^ Kudo et al. reported nationwide retrospective analysis of allogeneic HSCT in 30 children with refractory LCH in Japan. They showed that disease status at the time of HSCT was the most important prognostic factor. Thirteen patients died and long‐term OS was 57%. Death occurred in eight patients within 3 months after HSCT regardless of the intensity of conditioning regimens.^13^ In current study, clinical evaluation of both 2‐CdA/Ara‐C and HSCT is under investigation in LCH‐IV, international collaborative treatment protocol for children and adolescents with LCH.

There remains the need for a clearly defined third‐line chemotherapy for refractory and risk organ negative patients. Considering the effectiveness and feasibility of these treatment strategies, CLO monotherapy could be a therapeutic option besides other intensive chemotherapy regimens or allogeneic HSCT for treating refractory and/or recurrent LCH, as our two patients we presented here, although large‐scale studies are required to make a definitive conclusion.

## CONFLICT OF INTEREST

The authors have no conflict of interest in the publication of this paper.

## AUTHOR CONTRIBUTIONS

All authors had full access to the data in the study and take responsibility for the integrity of the data and the accuracy of the data analysis. *Conceptualization*, M.I. and Y.S.; *Investigation*, M.I. and Y.S.; *Resources*, M.I., T.N., S.K., T.S., K.M., Y.W., N.S., Y.S.‐N., M.O., T.R. and H.N.; *Data Curation*, M.I.; *Writing—Original Draft*; M.I.; *Writing—Review & Editing*; Y.S.; *Funding Acquisition*, Y.S.; *Supervision*, S.K.

## ETHICAL STATEMENT

The off‐label use of CLO was approved by the chemotherapy protocol committee of the Tohoku University Hospital. Informed consent was obtained from the guardians of two patients in this study.

## Data Availability

The data is available on request from the corresponding author.
